# Case of Poisoning by the Cashew Nut

**Published:** 1844-06-15

**Authors:** P. Le B. Stickney

**Affiliations:** Philadelphia


### CASE OF POISONING BY THE CASHEW NUT.

By P. Le B. Stickney, M. D., of Philadelphia.

James B——, a lad about sixteen years of age, was poisoned by rubbing upon the back of his hand, the acrid juice of the cashew nut.

The effects of the poison were first manifested by an excessive inflammation of the affected part, accompanied with pain and an almost intolerable itching.

This was followed by an eruption of small red pimples which soon suppurated, bearing a close resemblance to the pustular eruption produced by croton oil. In a short time, these pustules discharged a very small quantity of thin pus, coalesced, and became covered with a thin pellicle, filled with serum, giving to the skin the appearance of having been covered with small blisters.

The blistering or desquamation of the cuticle was confined to the part upon which the juice had been applied, excepting the lips, which being repeatedly rubbed by the hand presented a similar appearance, whilst the swelling and pustular eruption extended to the other parts of the body.

The penis and scrotum were enormously distended by an œdematous swelling, but the eruption was confined entirely to the scrotum.

The urine, which was voided in large quantities, was of a dark bottle-green colour, possessed its natural smell, and deposited no sediment. Unfortunately it was not analysed, and we have therefore no means of satisfactorily accounting for this peculiar colour.

The general health of the patient was not materially affected. On the first appearance of the swelling, there was some fever and thirst.

Saline purgatives, with warm and cold fomentations, and poultices of the slippery elm bark, were used with benefit.

A poultice of bread and milk, with the common plantain leaf, appeared to be most serviceable in removing the swelling and itching—perhaps the flaxseed poultice would have answered equally as well.

The cashew nut is a product of the Anacardium Occidentale, a small tree growing in the West Indies and other parts of tropical America.

The active property of the poison is found in the black juice contained between the outer and inner shell of the nut, and is exceedingly acrid and corrosive.

Mr. Worthington, of this city, some time since tried some experiments with the juice of the nut. Having dissolved it in æther, he obtained by evaporation a thick dark brown coloured oil, which contained the poisonous principle. He was deterred from further experiments on account of the severe effects produced upon him by the poison.

A more extended description of the plant and properties of the nut, may be found in the Appendix to the last edition of the U. S. Dispensatory, under the head of "Anacardium Occidentale."

